# Hospital-based Diagnosis of Hemorrhagic Fever, Encephalitis, and Hepatitis in Cambodian Children

**DOI:** 10.3201/eid0805.010236

**Published:** 2002-05

**Authors:** Y. Meng Chhour, Gaye Ruble, Rathavuth Hong, Kyi Minn, Yuvatha Kdan, Touch Sok, Ananda Nisalak, Khin Saw Aye Myint, David W. Vaughn, Timothy P. Endy

**Affiliations:** * National Pediatric Hospital, Phnom Penh, Cambodia; † Armed Forces Research Institute of Medical Sciences, Bangkok, Thailand; ‡ World Vision International, Phnom Penh, Cambodia; § Walter Reed Army Institute of Research, Silver Spring, Maryland, USA

**Keywords:** Cambodia, dengue, hemorrhagic fever, Japanese encephalitis, hepatitis, surveillance, Southeast Asia

## Abstract

Surveillance was conducted for three clinical syndromes (hemorrhagic fever, encephalitis, and hepatitis) in Cambodian children admitted to the National Pediatric Hospital in Phnom Penh from July 1996 through September 1998. Acute- and convalescent-phase sera, and cerebrospinal fluid, when applicable, underwent diagnostic evaluation for infections with *Dengue virus* (DENV), *Japanese encephalitis virus* (JEV), and Hepatitis A, B, C, and E viruses. Of 621 children admitted with hemorrhagic fever, 499 (80%) were confirmed to have either primary or secondary DENV infection. DENV rates were as high as 10.6/100 hospital admissions in September 1998. Of 50 children with clinical encephalitis, 9 (18%) had serologic evidence of JEV infection. Forty-four children had clinical hepatitis, most (55%) due to H*epatitis A virus* (HAV). One patient had *Hepatitis B virus*, and no patients had hepatitis C or E. This study identified a large number of children with vaccine-preventable diseases (JEV and HAV).

Infectious diseases continue to pose a major threat to populations in developing countries in tropical regions. Dengue is the most important arbovirus infection in Southeast Asia (*1*). It is spread by the bite of the vector mosquito, *Aedes aegypti*, and causes asymptomatic infection, mild to severe influenzalike symptoms (dengue fever), and plasma leakage and hemorrhage, which are sometimes fatal (dengue hemorrhagic fever). In 1995, Rathavuth et al. conducted 2 days of surveillance for hemorrhagic fever in children admitted to the National Pediatric Hospital (NPH) in Phnom Penh (*2*). Their findings of a high frequency of secondary *Dengue virus* (DENV) infection, a low mean age of admission, and the presence of all four dengue serotypes suggested that dengue was highly endemic in Cambodia.

Bacterial, viral, fungal, and parasitic agents are all causes of encephalitis or encephalopathy in children in Southeast Asia. Tsai reported that the main causes of encephalitis in rural Asia included tuberculosis, typhoid fever, cerebral malaria, and viruses such as DENV, herpes simplex, measles*, Enterovirus*, and HIV (*3*). Few reports have been published about the incidence or possible causes of encephalitis in Cambodian children. In a report by Sunara et al., surveillance at two pediatric hospitals in Phnom Penh from 1990 through 1994 showed >300 cases of acute encephalitis in children (*4*). While the cause for many of these cases was suspected to be *Japanese encephalitis virus* (JEV), laboratory confirmation was lacking.

Only one report has discussed the prevalence of markers for Hepatitis viruses A, B, and C (HAV, HBV, HCV), in Cambodia. In 1991, Thuring et al. conducted a serologic study for markers of viral hepatitides and HIV in Takeo, a southern province (*5*). In that study adults and children, both healthy and those ill with liver or kidney disease, were examined. HAV was the most frequent cause of acute hepatitis in these children, occurring in 11 (32%) of 34 pediatric patients. Ongoing infection with HBV accounted for 18%, and one child tested positive for HCV-specific antibody. That study did not screen for *Hepatitis E virus* (HEV) infection.

Our study was undertaken to characterize the extent of disease in Cambodian children, specifically for the following three syndromes: hemorrhagic fever (HF), encephalitis, and hepatitis.

## Materials and Methods

Surveillance was conducted at NPH in Phnom Penh. This hospital, one of two pediatric referral hospitals in Cambodia, serves a population of approximately 2.7 million children ages <14. Enrolled in this study were children admitted with any of the following clinical signs: HF (fever, headache, or rash, and on physical examination, a positive tourniquet test, ascites, pleural effusion bleeding, or shock); encephalitis (headache, fever, or neck stiffness, and alteration of consciousness or focal neurologic signs); and hepatitis (lethargy, anorexia, nausea or vomiting, abdominal pain, hepatomegaly, scleral icterus, or jaundice). Case definitions were kept broad to capture as many cases as possible.

On the basis of published criteria from the World Health Organization, cases of DHF were classified into one of four grades of severity. Grade 1 includes fever with nonspecific symptoms; the only hemorrhagic manifestation is a positive tourniquet test, easy bruising, or both. Grade 2 includes Grade 1 manifestations plus spontaneous bleeding (usually skin hemorrhages). Grade 3 includes circulatory failure (rapid, weak pulse, hypotension) and cold, clammy skin. Grade 4 is manifested by profound shock with undetectable blood pressure or pulse. The last two grades are considered to be dengue shock syndrome (DSS).

Sera were collected on the day of admission, at the time of discharge, and, in some cases, on follow-up exam. However, due to the nature of the population, a follow-up visit was not always possible, and therefore diagnosis relied on only an admission and discharge sample. Sera were stored at -70°C until transported to the Armed Forces Research Institute of Medical Sciences in Bangkok, Thailand, on dry ice. Clinical criteria for admission diagnosis directed the subsequent diagnostic workup. All DHF and encephalitis cases were tested for both JEV and DENV. Sera were tested for immunoglobulin (Ig) G and IgM antibody against DENV and JEV by use of an antibody-capture enzyme-linked immunoassay (EIA) and previously published criteria of acute and primary or secondary dengue (*6*). Virus isolation was attempted with acute-phase serum specimens, as previously described (*7*).

Sera were screened for IgM antibody to HAV, IgM antibody to hepatitis B core antigen (HbcAg), hepatitis B surface antigen (HbsAg), and total Ig to HCV by using commercially available kits (HAVAB-EIA, Corzyme-M, AUZYME Monoclonal, and HCV EIA Third Generation; Abbott Laboratories, Abbott Park, IL). Assays were performed as recommended by the manufacturer.

All samples were tested for total Ig and IgM to HEV by an indirect second-generation EIA developed at the Department of Virus Diseases, Walter Reed Army Institute of Research. The assay quantifies total Ig and IgM reactive with recombinant HEV capsid protein expressed using a baculovirus system expressed in U/mL (*8*). To control interassay variation, all specimens were tested in duplicate wells, with all specimens from a single patient tested together on the same plate. A patient was considered to have HEV infection if there was virologic (HEV RNA positive) or serologic (IgM >100 U/mL, total Ig >500 U/mL) evidence of acute infection.

### Statistics

All statistical procedures were performed by using SPSS for Windows, Version 10.0 (SPSS Inc., Chicago, IL). Fisher’s exact test (two-tailed) was used to determine significant difference in the number of boys with encephalitis and diagnosed as having JEV compared with girls with the same syndrome and final diagnosis.

## Results

**Figure 1:**
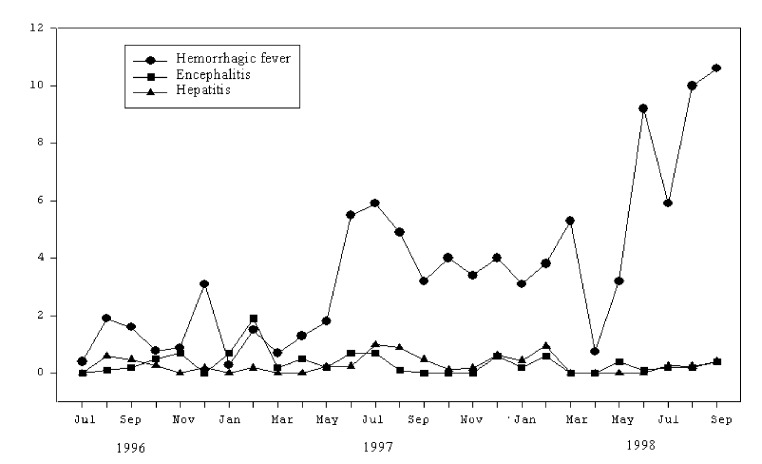
Admission rates by disease syndrome and month, National Pediatric Hospital, 1996–1998.* *Rates are given as number of cases per 100 hospital admissions.

**Figure 2:**
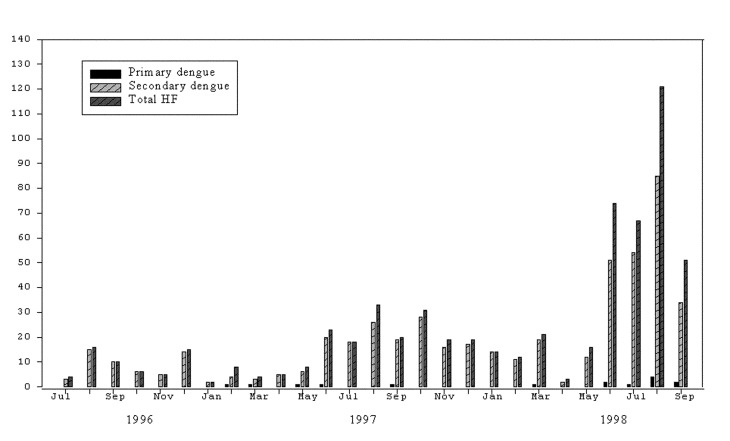
Cases of hemorrhagic fever (HF), secondary and primary dengue, National Pediatric Hospital, 1996–1998.

Figure 1 shows the rates of HF, encephalitis, and hepatitis per 100 hospital admissions, by month and year. From July 1996 through September 1998**,** 621 children were admitted with a diagnosis of HF (Table 1). Of those, 495 were confirmed to have a secondary DENV infection by serologic tests; 14 had primary dengue. Figure 2 illustrates the number of confirmed DENV patients compared with the total number of patients with HF.

**Table 1:** Characteristics of pediatric patients with clinical hemorrhagic fever, encephalitis, or hepatitis, National Pediatric Hospital, Phnom Penh, Cambodia, July 1996–September 1998^a^

Syndrome	Total no. of cases	**Males**	Females	Mean age (range)	Outcome
Total hemorrhagic fever	621	288	332	7 years (5 mo-15 yrs)	11^b^
Secondary dengue	495	222	272	7 years (8 mo-15 yrs)	4^b^
Primary dengue	14	8	6	4 years (5 mo-9 yrs)	—
Total encephalitis	50	15	35	4 years (3 mo-14 yrs)	17^b^
					12^c^
JE	9	6	3	6 years (3-10 yrs)	2^b^
					2^c^
Total hepatitis	44	21	23	6 years (2 mo-14 yrs)	—
Hepatitis A	24	12	12	5 years (2 mo-9 yrs)	—
Hepatitis B	1	1	0	10 years	—

^a^Total number of hospital admissions during this period was 16,492 children. ^b^Deaths. ^c^Disabled. JE, Japanese encephalitis; —, all recovered.

The severity of DHF can be classified into four grades based on two pathophysiologic findings: hemorrhage and shock. In this study, 41 of the 509 total confirmed dengue patients had DHF Grade 1; 145 patients had DHF Grade 2; 180 patients had DHF Grade 3; and 29 patients had DHF Grade 4.

Of the 75 samples tested, 22 were polymerase chain reaction (PCR)-positive for virus. DENV-2 was isolated from 14 samples, DENV-3 from seven samples, and DENV-4 from one sample. All three serotypes were found in children living in Phnom Penh. DENV-2 and -3 were found in Kampong Speu and Kampong Cham. The other provinces appeared to have only one circulating dengue serotype, based on the small number of positive samples.

**Figure 3:**
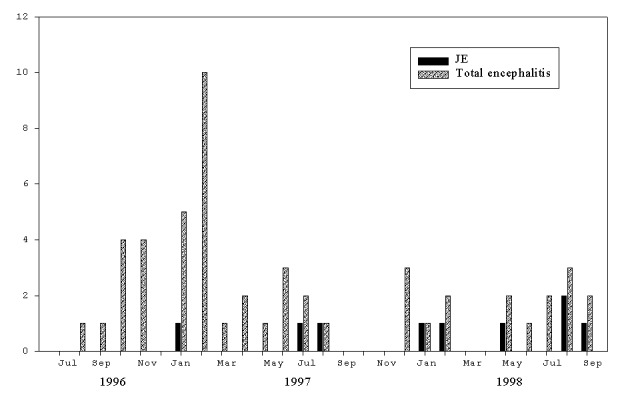
Total number of cases of encephalitis versus Japanese encephalitis, National Pediatric Hospital, 1996–1998.

During this same period, 50 children were admitted to NPH with a diagnosis of encephalitis; 9 (18%) were due to JEV (Table 1) and 2 to acute secondary dengue infection. There was no evidence of concurrent infections. Figure 3 illustrates the number of cases of JEV, by month and year, compared with the total encephalitis cases. While over twice as many girls as boys had this syndrome, significantly more boys were diagnosed with JEV infection (p=0.015). The final outcome for children seen at NPH with encephalitis was poor: death or disability occurred in 29 (58%) of the children.

**Figure 4:**
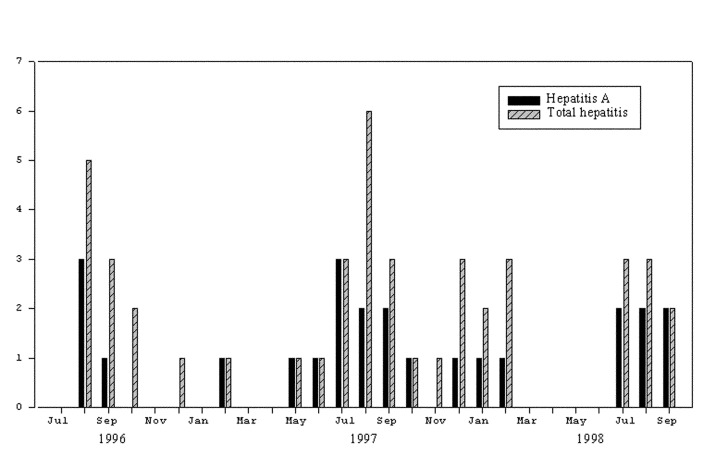
Total number of cases of hepatitis versus hepatitis A, National Pediatric Hospital, 1996–1998.

Forty-four children were admitted with a diagnosis of hepatitis. Twenty-four cases were confirmed to be due to acute HAV infection on the basis of elevated HAV IgM antibodies (Table 1). One patient had serologic evidence of acute hepatitis B, and no serologic evidence for HCV or HEV infections. Figure 4 illustrates the number of HAV patients compared with the total number of patients admitted to NPH with a clinical diagnosis of hepatitis. Of the 24 children hospitalized with hepatitis A, 17 (71%) had onset in July, August, and September. Most children admitted to NPH came from Phnom Penh (339 patients) or its adjacent province, Kandal (146 patients). Table 2 shows the distribution of inpatients by province and syndrome on admission.

**Table 2:** Distribution of inpatients by most widely represented provinces, National Pediatric Hospital, Cambodia

Syndrome upon admission	No. (%) of patients by province						
	Phnom Penh	Kandal	Kampong Speu	Kampong Cham	Takeo	Prey Veng	Total
Hemorrhagic fever	309 (50)	116 (19)	9 (3)	40 (6)	20 (3)		494 (81)
Encephalitis	15 (30)	15 (30)	8 (16)		5 (11)		43 (87)
Hepatitis	15 (34)	15 (34)	3 (7)	3 (7)		5 (11)	41 (93)
Total	339	146	20	43	25	5	

## Discussion

This surveillance was undertaken to characterize the extent of disease in Cambodian children with respect to three specific syndromes: hemorrhagic fever, encephalitis, and hepatitis. To our knowledge, this is the first such study conducted in Cambodia. As in other Southeast Asian countries, DHF accounted for a large percentage of hospitalizations and deaths of Cambodian children. Dengue was confirmed in 82% of children admitted to NPH with symptoms that suggested dengue fever. Serologic results for the remaining 112 HF cases suggested DENV infection in most instances, but lack of a convalescent-phase sample prevented definitive diagnosis. DHF has been reported as a leading cause of hospitalization and death of children throughout Asia in 1998 (*9*). Our surveillance shows that in August and September 1998, the hospitalization rate for children with HF exceeded 10 per 100 hospital admissions.

Similarly, Japanese encephalitis has been reported to occur in nearly every country in Asia (*10*). While the disease is presumed to be endemic in Cambodia, no laboratory-confirmed data on disease frequency have been published until now. During this 2-year study, 18% of children admitted to NPH with encephalitis had JEV infection. Similar to reports elsewhere (*11*), more cases of JEV infection occurred in boys than in girls (six versus three, respectively). Transmission of disease is usually seasonal, from late summer to early fall. In our study, 67% of cases were reported from May to October; the remainder occurred in January or February.

HAV infection is highly endemic in developing countries that lack adequate clean water and have poor sanitary conditions (*12*). According to the 1998 Cambodian General Population Census, only 29% of the population has access to safe water (range 23.7%-60.3%) (*13*). In poorer countries, most children develop antibodies to HAV by age 10. A seroprevalence study conducted on 200 healthy Cambodian children from 1990 through 1991 showed that 97% were positive for anti-HAV IgG by 15 years of age (*5*). While hepatitis A is largely considered a subclinical infection in children in Thuring’s study, it accounted for 32% of acute hepatitis in hospitalized children in our study; hepatitis A accounted for 55% of the pediatric patients hospitalized with suspected hepatitis.

One (2%) of 44 children showed evidence of ongoing HBV infection. This contrasts with Thuring’s earlier study, in which 18% of children with acute hepatitis were actively infected with HBV (HbsAg positive). However, similar to Thuring’s study, we saw no evidence of HCV in this population.

No indication of acute hepatitis E was found in our study, nor did any of the children admitted to NPH with hepatitis have evidence of prior exposure to HEV. Similar to hepatitis A, hepatitis E is common in countries that lack adequate clean water and in which general sanitation is poor. In Southeast Asia, epidemics of HEV have been reported in Myanmar, Vietnam, and Indonesia (*14*). In most disease-endemic areas, up to 5% of the children are positive for anti-HEV antibodies. While clinical attack rates of hepatitis E are reported to be highest in young adults (15-40 years), a recent report from India noted anti-HEV antibodies in >60% of children <5 years old (*15*). Cambodia is surrounded by countries (except Thailand) that are endemic for HEV; therefore, the total lack of anti-HEV IgG in this Cambodian population was unexpected.

Currently, Cambodian children are given BCG, polio, diphtheria-tetanus-pertussis, and measles vaccines, although coverage varies throughout the country. Instituting childhood HAV immunizations could benefit countries that have shown a decrease in age-related HAV seroprevalence concomitant with improved socioeconomic development (*16*,*17*). While the seroprevalence for HAV in Thuring’s 1991 study approached 100% in children by age 15, no HAV seroprevalence data have been gathered since then. Further studies are warranted to determine if additional vaccines, such as those for HAV and JEV, should be added to the nation’s immunization program.
